# Monthly Trajectories of Mood Improvement Following a Workplace Mindfulness Programme: A Randomised Controlled Pilot Trial

**DOI:** 10.7759/cureus.89929

**Published:** 2025-08-12

**Authors:** Shinyu Kise, Shohei Yoshihara

**Affiliations:** 1 Department of Tourism and Health Studies (sponsored by Ryukyuseimeisaiseikai), Institute for Tourism and Health, Naha, JPN; 2 TEAM PTRD JAPAN®, F-CUBE Japan Inc., Tokyo, JPN

**Keywords:** mindfulness, mood disturbance, poms2, randomized controlled trial, workplace

## Abstract

Background: Mindfulness‑based interventions (MBIs) are widely adopted to mitigate workplace stress, yet their month-by-month impact on mood has rarely been quantified.

Methodology: A total of 25 full‑time Japanese employees (60% men; mean ± SD age 40.1 ± 6.2 years) were randomised to a three‑month MBI arm (n = 12) or a wait‑list control arm (n = 13). The MBI comprised three 90‑min workshops (Weeks 0, 4, 8) plus 10‑min daily self‑practice. Measuring Total Mood Disturbance scores (TMD; POMS‑2‑SF) was the primary outcome; fatigue (100‑mm VAS) and presenteeism (Stanford Presenteeism Questionnaire; SPQ) were secondary. Assessments occurred at baseline and monthly for three months. Linear mixed‑effects models with participant random intercepts tested group × time interactions; 95% bootstrap CIs (1,000 iterations) were generated.

Results: TMD declined cumulatively in the MBI arm (‑4.2, ‑4.4, ‑6.3 points at Months 1‑3), while controls rose +1.0 point; interaction β = ‑2.56 points·month⁻¹ (95 % CI ‑4.31 to ‑0.71, p = 0.014). Fatigue (p = 0.47) and presenteeism (p = 0.41) trends favoured the intervention but were non‑significant.

Conclusions: A low‑dose workplace MBI produced additive three‑month reductions in mood disturbance. Monthly assessments clarify change dynamics and justify larger confirmatory trials integrating physiological and organisational endpoints.

## Introduction

Occupational stress is a major driver of absenteeism and presenteeism worldwide. A systematic review showed that mindfulness‑based interventions (MBIs) improve employee mental health across diverse industries [1]. Pioneering workplace studies, including the RCT by Wolever et al. [2] and the experience‑sampling study by Hülsheger et al. [3], demonstrated significant post‑programme stress reduction; however, most studies measured outcomes only at baseline and immediately after the intervention, leaving the temporal trajectory of change unclear. To address this gap, we mapped monthly mood changes over three months following a workshop‑based, low‑dose MBI delivered to Japanese employees.

## Materials and methods

Study design and participants

The study comprised a parallel‑group randomised controlled trial (RCT) conducted in accordance with CONSORT‑NP guidelines. The inclusion criteria involved≥20y, full‑time employment, and those with diagnosed major psychiatric disorders were excluded. A total of 25 volunteers were block‑randomised (block=4) to the MBI or wait‑list control groups.

Intervention

Instructor‑Led Workshop Timetable

The programme combined three instructor‑led 90‑minute workshops delivered once every four weeks (Weeks 0, 4, 8) with daily home practice (10 minutes, twice per day) for eight weeks (Table 1).

**Table 1:** Instructor‑Led Workshop Timetable min = minutes; ANS: autonomic nervous system; HRV = heart‑rate variability; HIIT = high‑intensity interval training

Component	Workshop 1 (Week 0)	Workshop 2 (Week 4)	Workshop 3 (Week 8)
Mindfulness practice	Breathing meditation, 5 min	–	Body‑scan meditation, 10 min
Psycho‑education	Well‑being science, 5 min; sleep hygiene, 10 min; physical‑activity guidelines, 5 min; sedentary‑behaviour risks, 5 min; economic burden of musculoskeletal pain, 5 min; ANS basics, 5 min	Importance of gut health, 15 min; gut microbiota, 15 min; diet advice, 10 min; food additives, 5 min; artificial sweeteners, 5 min; brain–gut axis, 5 min; fasting basics, 5 min	HIIT theory, stress physiology and HRV, 5 min
Somatic / movement practice	Self‑stretch (ANS balancing), 10 min; pair stretch, 5 min	Pair stretch, 5 min; gut‑massage self‑release, 5 min	Self‑massage, 15 min; lower‑limb stretch, 10 min; pair stretch, 5 min; HIIT circuit, 5 min
Interactive dialogue	Group sharing, 15 min; Q&A, 10 min; take‑home goals, 10 min	Reflections on first workshop, 10 min; Q&A, 5 min; take‑home goals, 5 min	Mindful listening, 10 min; open dialogue, 15 min; Q&A, 10 min; summary, 5 min

Home Practice Materials and Recommendations

Daily self-practice: Participants were instructed to engage in daily self-practice, consisting of 5 minutes of stretching and 5 minutes of mindful breathing meditation, each performed once in the morning and once in the evening. Additionally, participants were recommended to incorporate regular physical activities, including squats and high-intensity interval training (HIIT), into their routines. Dietary recommendations emphasised a diet primarily based on Japanese-style and paleo-style meals, along with a suggestion to undertake a one-time fasting session. Participants were also advised to aim for a daily physical activity target of 8,000 steps.

Participants used the Stress Scan app (Stress Scan Inc., Tokyo, Japan) daily to monitor their autonomic nervous system balance, providing visual biofeedback to help track stress levels and enhance awareness of their physiological state. Participants were instructed to perform measurements immediately after their daily self-practice.

Outcomes and data collection

Primary Endpoint

The primary endpoint was to measure Total Mood Disturbance scores using the Profile of Mood States-2 Short Form (POMS-2-SF).

Secondary Endpoints

Secondary endpoints included fatigue assessed using a 100-mm visual analogue scale and presenteeism evaluated with the Stanford Presenteeism Questionnaire (SPQ). Assessments were conducted at baseline and monthly for three months. Instrument reliability, scoring and a full statistical analysis plan were pre‑specified in the protocol.

Statistical Analysis

Analyses used Python 3.11.3 with pandas and statsmodels packages. Missing values (4.7%) were multiply imputed (m = 5, random‑forest). Linear mixed‑effects models included fixed effects for group, time (categorical), and their interaction, with participant random intercepts. Two‑tailed p < 0.05 denoted significance; a clinically important difference was ≥ 0.5 SD of baseline scores.

Ethical Considerations

The study was approved by the Ryusei Hospital Ethics Committee (approval No. 2024‑02; 25 Dec 2024). All participants provided written informed consent. This study is registered at ClinicalTrials.gov (NCT07029022).

## Results

Participant flow and baseline characteristics

**Figure 1:**
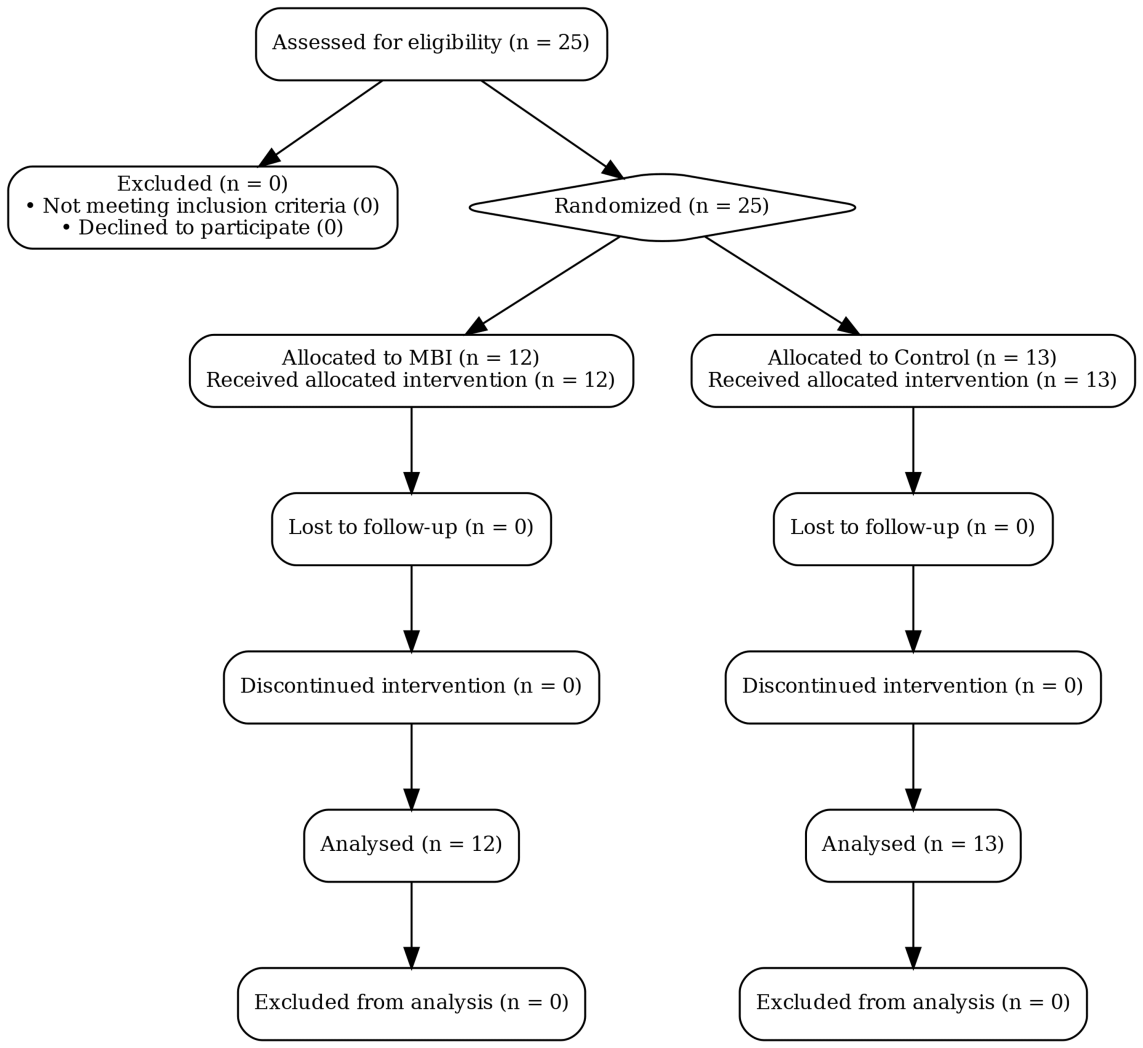
CONSORT‑NPT Participant Flow Diagram The flow diagram illustrates participant progression from recruitment through randomisation, follow-up, and analysis. A total of 25 employees meeting the eligibility criteria were enrolled and subsequently randomised by an independent third party using a computer-generated random sequence with a 1:1 allocation ratio (block size = 4). Allocation concealment was ensured using sealed envelopes prepared externally in advance, opened only after participant enrollment. All participants received the allocated interventions and completed all follow-up assessments. Analysis was conducted according to the intention-to-treat (ITT) principle, with no participants lost to follow-up or excluded from the analysis. Outcome data were self-reported by participants via Google Forms and subsequently exported by a blinded principal investigator, with treatment group identifiers masked.

All 25 employees screened met the inclusion criteria and were randomised (Figure 1). Table 2  confirms baseline comparability: mean age was approximately 40 years in both groups (p=0.88) and sex distribution was balanced (p=0.87). Baseline Total Mood Disturbance (TMD) scores did not differ significantly (p=0.62), indicating successful randomisation.

**Table 2:** Baseline Characteristics Values are mean ± SD or n (%). The Test statistic column shows t‑values (Student’s t‑test) or χ²‑values (Pearson’s χ²‑test) as appropriate. Two‑sided p < 0.05 was considered statistically significant.

Variable	MBI (n = 12)	Control (n = 13)	Test statistic	p
Age, y	40.3 ± 6.1	39.9 ± 6.4	t(23) = 0.16	0.88
Male	7 (58 %)	8 (62 %)	χ²(1) = 0.03	0.87
Baseline TMD, pts	15.6 ± 6.3	16.8 ± 7.1	t(23) = 0.50	0.62

Primary Outcome: Total Mood Disturbance

The linear mixed‑effects model revealed a significant group × time interaction (β = −2.56 points·month⁻¹, t= −2.79, 95% CI −4.31 to −0.71, p=0.014). As detailed in Table 3, the mindfulness‑based intervention (MBI) group showed a progressive decline in TMD (−27%, −28%, and −40% at Months 1-3), whereas the control group experienced a slight increase (+7 %). The between‑group difference became statistically significant at Month 1 (Δ = −3.3 points, p=0.041) and widened to −8.7 points by Month 3, corresponding to a large effect size (Cohen’s d ≈ 1.25).

**Table 3:** Monthly Trajectory of TMD TMD: Total Mood Disturbance; Δ = unadjusted between‑group mean difference. LMM t = t‑value from the linear mixed‑effects model for the group × time interaction (participant random intercepts). Two‑sided p < 0.05 deemed significant; † indicates p < 0.05.

Month	MBI Mean ± SD	Control Mean ± SD	Δ (MBI − Ctrl)	LMM t	p
0	15.6 ± 6.3	16.8 ± 7.1	−1.2	–	–
1	11.4 ± 5.7	14.7 ± 7.4	−3.3	−2.15	0.041 †
2	11.2 ± 5.1	15.0 ± 7.8	−3.8	−2.40	0.023 †
3	9.3 ± 4.9	18.0 ± 8.2	−8.7	−2.79	0.014 †

Secondary Outcomes

Table 4 shows that fatigue decreased by 13 mm in the MBI arm but rose 2 mm in controls; however, the interaction did not reach significance (p=0.47). Presenteeism improved by 3% in the MBI arm and declined by 3% in controls (p=0.41). Although non‑significant, both trends favoured the intervention and aligned with the direction of the primary outcome.

**Table 4:** Secondary Outcomes at Baseline and Month 3 Values are mean ± SD. LMM t = t‑value from the linear mixed‑effects model (group × time interaction). Two‑sided p < 0.05 considered significant.

Outcome	Baseline MBI	Baseline Control	Month 3 MBI	Month 3 Control	LMM t	p
Fatigue VAS (mm)	55 ± 15	56 ± 16	42 ± 12	58 ± 16	−0.73	0.47
SPQ (%)	78 ± 7	77 ± 8	81 ± 5	74 ± 9	−0.83	0.41

Adherence and Safety

Attendance was perfect for all three workshops, and no adverse events were reported. The absence of drop‑outs strengthens the internal validity of the findings.

## Discussion

Principal findings

The intervention produced a cumulative 8.7‑point reduction in TMD, exceeding the half‑standard‑deviation benchmark for clinical relevance [4] and approaching the effects reported for higher‑dose protocols such as the eight‑week programme evaluated by Wolever et al. [2] and the online dose‑response study by Aikens et al. [5]. Improvement was already evident at Month 1, suggesting that even limited instructor contact can yield early psychological benefits that may reinforce subsequent engagement.

Comparison with previous research

Dose‑response work indicates that greater home‑practice time predicts larger gains [5]. Most corporate RCTs still rely on weekly instructor‑led sessions lasting six to eight weeks [6-8]; our “once‑monthly + daily micro‑practice” model achieved comparable mood benefits with substantially fewer contact hours.

Our non‑significant fatigue and presenteeism outcomes resemble those of Chin et al. [9], implying that behavioural and productivity gains may lag behind affective change. Tailored mindfulness programmes for resident physicians have likewise reduced distress and enhanced perceived care quality [10]. A workplace‑focused systematic review of healthcare professionals reported consistent psychological benefits [11], and a subsequent review emphasised similar gains among intensive‑care nurses [12].

Stress‑focused meta‑analytic evidence from 23 workplace RCTs shows medium pooled effects on psychological outcomes, while effects on somatic measures remain less clear [13]. Brief smartphone applications for healthcare staff [14], physiological studies demonstrating reduced cortisol and increased heart‑rate variability in healthy adults [15], and guided internet‑ and mobile‑based programmes for employees [16] collectively suggest that digital formats can broaden access without sacrificing efficacy. Rigorous economic evaluations are scarce; the only workplace cost‑utility analysis to date did not demonstrate cost‑effectiveness within a two‑year horizon [17]. A broader meta‑analysis of healthy populations confirms medium‑sized benefits of mindfulness‑based stress reduction [18].

Mechanistic considerations

Improvements in decentering and cognitive re‑appraisal - core skills explicitly cultivated during mindfulness practice - likely underpin the observed mood gains, whereas somatic or productivity changes may require longer practice duration or higher cumulative “dose”.

Strengths and limitations

Strengths include 100% retention, preregistration, rigorous mixed‑effects modelling with bootstrap confidence intervals, and minimal contact hours that enhance scalability. Limitations include (1) a small sample size, (2) exclusive reliance on self‑report measures, (3) absence of an attention‑matched control, and (4) lack of biomarker or objective productivity endpoints.

## Conclusions

Three brief workshops plus daily micro‑practice yielded clinically meaningful, cumulative mood improvements over three months. Future multi‑site trials should incorporate biomarkers, organisational metrics, and cost‑utility analyses while comparing hybrid and fully digital delivery modes to optimise reach and efficiency.
